# Sex-Specific Contrasting Role of BECLIN-1 Protein in Pain Hypersensitivity and Anxiety-Like Behaviors

**DOI:** 10.1523/ENEURO.0244-24.2024

**Published:** 2025-02-03

**Authors:** Fariya Zaheer, Gabriel J. Levine, Ana Leticia Simal, Seyed Reza Fatemi Tabatabaei, Tami A. Martino, Giannina Descalzi

**Affiliations:** ^1^ Department of Biomedical Sciences, University of Guelph, Guelph, Ontario N1G 2W1, Canada; ^2^ Center for Cardiovascular Investigations, University of Guelph, Guelph, Ontario N1G 2W1, Canada

**Keywords:** anxiety, autophagy, BECLIN-1, female, neuropathic pain, sex differences

## Abstract

Chronic pain is a debilitative disease affecting one in five adults globally and is a major risk factor for anxiety ( Goldberg and McGee, 2011; Lurie, 2018). Given the current dearth of available treatments for both individuals living with chronic pain and mental illnesses, there is a critical need for research into the molecular mechanisms involved in order to discover novel treatment targets. Cellular homeostasis is crucial for normal bodily functions, and investigations of this process may provide better understanding of the mechanisms driving the development of chronic pain. Using the spared nerve injury (SNI) model of neuropathic pain, we found contrasting roles for BECLIN-1 in the development of pain hypersensitivity and anxiety-like behaviors in a sex-dependent manner. Remarkably, we found that male SNI mice with impaired BECLIN-1 function demonstrated heightened mechanical and thermal hypersensitivity compared with male wild-type SNI mice, while female SNI mice with impaired BECLIN-1 function demonstrated similar thresholds to the female wild-type SNI mice. We also found that disruptions of BECLIN-1 prevented SNI-induced increases in anxiety-like behaviors in male mice. Our data thus indicate that BECLIN-1 is differentially involved in the nociceptive and emotional components of chronic pain in male but not female mice.

## Significance Statement

One in five adults suffers from chronic pain, and it is a major risk factor for anxiety. Close to three-quarters of the population suffering from chronic pain are women, yet the vast majority of preclinical research uses solely male models, and excludes females. In this manuscript, we use female and male mice to discover a novel role for BECLIN-1 in neuropathic pain and comorbid anxiety-like behaviors in mice. We found that disruptions of *Beclin-1* reduce nociceptive hypersensitivity while preventing pain-induced increases in anxiety-like behaviors. Notably, these effects were sex-dependent, where only males, but not females, showed BECLIN-1-mediated effects. Our data thus indicate that macroautophagy is differentially involved in nociception and anxiety in male, but not female, mice.

## Introduction

Chronic pain is a debilitative disease affecting one in five adults globally and is a major risk factor for anxiety (Goldberg and McGee, 2011; Lurie, 2018). Chronic neuropathic pain is a debilitating condition that is often resistant to currently available treatments. Neuropathic pain develops upon direct and/or indirect injury to peripheral nervous tissue (Xu et al., 2012). In addition to pain hypersensitivity, over 60% of people with chronic pain experience severe depression and anxiety (Bair et al., 2008; Choinière et al., 2010). Disruptions of homeostasis within the central nervous system, which can be induced by traumatic nerve injury, can activate cellular processes that can promote long-term changes in neuronal activity. A critical mechanism involved in homeostasis is the process of autophagy, which ensures appropriate disposal of damaged intracellular organelles to maintain its equilibrium (Glick et al., 2010). BECLIN-1 plays a major role in the process of macroautophagy (herein autophagy) through its interaction with PI3K complexes and is responsible for the delivery of autophagosomes to lysosomes for intracellular degradation, promoting the restoration of cellular homeostasis (Kang et al., 2011). Notably, impaired function of BECLIN-1 protein leads to a deficiency in autophagy (He et al., 2012), and reductions in autophagy have been shown to alter inflammatory responses, such as interleukin-1 beta (IL-1β) release (Houtman et al., 2019) and impaired clearance of cellular debris. Numerous animal models of neuropathic pain have consistently shown IL-1β expression in numerous regions, including injured peripheral nerves, dorsal root ganglia, and the dorsal horn of the spinal cord (Scholz and Woolf, 2007). This prompts the possibility that BECLIN-1 may play a critical role in chronic pain pathogenesis and pathophysiology. Indeed, a recent study showed that transgenic knockdown of *Beclin-1* enhanced nociceptive hypersensitivity in male mice injected with complete Freund's adjuvant, a model for chronic inflammatory pain (Tam et al., 2024). Moreover, reductions in autophagy within the prelimbic cortex have been implicated in neuropathic pain-induced anxiety-like behaviors in male rats (Fu et al., 2024). These findings suggest a potential role for BECLIN-1-mediated autophagy in nociceptive and anxiety-related consequences of neuropathic pain. In this study, we used the spared nerve injury (SNI) model of neuropathic pain to determine the role of BECLIN-1 in chronic pain development in male and female mice (Decosterd and Woolf, 2000; Cichon et al., 2018). Using the transgenic mouse line, *Bcl2^AAA^*, which is defective for autophagy due to knock-in mutations in critical phosphorylation sites (Thr69Ala, Ser70Ala, and Ser84Ala) that prevent the dissociation of BECLIN-1 from Bcl-2, thereby inhibiting autophagy (He et al., 2012), we found that the male *Bcl2^AAA^* mice show enhanced SNI-induced pain hypersensitivity when compared with wild-type (WT) mice, whereas female *Bcl2^AAA^* SNI mice showed similar thresholds compared with female WT SNI mice. Furthermore, we found that SNI increases anxiety-like behaviors in WT mice, while male *Bcl2^AAA^* mice exposed to SNI behaved similarly to sham-treated controls. In contrast, we found that female *Bcl2^AAA^* mice exposed to SNI displayed similar anxiety-like behavior to WT SNI mice. This indicates that BECLIN-1 may prevent pain hypersensitivity and promote pain-induced increases in anxiety-like behaviors in male mice, but not in female mice.

## Materials and Methods

### Animals

We used male and female adult (11–12 weeks old) wild-type (WT) C57BL/6NCrl (Charles River Laboratories) and *Bcl2^AAA^* mice (B6.129X1(Cg)-*Bcl2^tm1.1Sjk^*, The Jackson Laboratory strain #018430) for all experiments. *Bcl2^AAA^* transgenic mice are bred homozygous × homozygous, with the suggested control being C57Bl/6 mice. In terms of derivation, a targeting vector containing an frt-flanked puromycin resistance cassette was used to insert alanine substitutions into amino acid residues 69 (threonine), 70 (serine), and 84 (serine) of the *Bcl2* gene (Bcl2AAA). The alanine substitutions were directed to three conserved phosphorylation sites of the exon 2 loop region. The construct was electroporated into 129X1/SvJ-derived RW4 embryonic stem (ES) cells. Correctly targeted ES cells were injected into C57BL/6 blastocysts, and resulting chimeric mice were bred with B6;SJL-Tg(ACTFLPe)9205Dym/J to remove the puro cassette. Mice were backcrossed to C57BL/6 for >10 generations. For all mice, sex was determined through anogenital distancing. All mice were housed in threes with a 12 h light/dark cycle (7:00 on, 19:00 off), in a room with an ambient temperature of 21–24°C with access to food and water provided *ad libitum*. All cages were provided with environmental enrichment, consisting of Crink-l’Nest bedding (Lab Supply), a Kimwipe, a polycarbonate igloo, and an elevated polycarbonate loft suspended from the cage roof. All animal procedures were performed in accordance with the University of Guelph's animal care committee's regulations.

### Transgenic mouse line

The *Bcl2^AAA^* mice contain knock-in mutations in three amino acid phosphorylation sites, at position T69A, S70A, and S84A. The construct was electroporated into 129X1/SvJ-derived RW4 embryonic stem (ES) cells. Correctly targeted ES cells were injected into C57BL/6 blastocysts, and resulting chimeric mice were bred with B6;SJL-Tg(ACTFLPe)9205Dym/J to remove the puro cassette. Mice were backcrossed to C57BL/6 for >10 generations. This prevents stimulus-induced disruption of the BCL2- BECLIN-1 complex, and in doing so, this prevents the activation of autophagy (He et al., 2012).

### Neuropathic pain model

The spared nerve injury (SNI) model was used as previously reported (Decosterd and Woolf, 2000; Descalzi et al., 2017; Cichon et al., 2018). Briefly, anesthesia was induced via inhaled anesthetic (5% isoflurane) and maintained at 1–3% isoflurane throughout surgery. A 1 cm skin incision was made on the left hindleg at midthigh level, followed by blunt dissection of the muscle layers, revealing the sciatic nerve and its three branches. The common peroneal and tibial nerves were carefully ligated with an 8.0 Vicryl suture (Ethicon, Johnson & Johnson) and transected, and a 1–2 mm section of each of these nerves was removed. The sural nerve was left intact. The skin was then closed and stitched with 5.0 Prolene sutures (Ethicon, Johnson & Johnson). Sham-operated mice were exposed to the same procedure, but all nerves were left intact.

### Mechanical threshold assessment

Mechanical thresholds were determined through von Frey filaments using the up-and-down method (Chaplan et al., 1994; Deuis et al., 2017). All mice were habituated to the testing apparatus which consisted of a plexiglass container, with an elevated wire mesh flooring for 2 h for 2 consecutive days, and baseline testing occurred on Day 3. Following the surgery, von Frey tests took place on Days 3, 7, 14, 21, and 28 postsurgery, after habituating for 40 min on each testing day. Pain thresholds were recorded after pain responses were observed 50% of the time per stimulus. Experiments were performed by a researcher blind to treatment and genotype. Measures of SNI-induced allodynia were recorded as previously reported (Sorge et al., 2015; Tam et al., 2024). Percent allodynia was assessed as a percentage of the maximum possible allodynia using the formula: percentage allodynia = [(baseline threshold − post-SNI threshold) / baseline threshold] × 100. Total allodynia intensity was then determined using the area under the curve using the trapezoid rule, over the post-SNI testing period as previously reported (Samuelsson et al., 2005).

### Thermal threshold measures

Thermal pain sensitivity was assessed using the hot plate tests (Bioseb) as previously described (Descalzi et al., 2013; Deuis et al., 2017). Briefly, mice were placed on a plate heated to 50 ± 0.2°C, and the latency to withdraw, flick, or lick the hind paws was recorded. Each trial was limited to 30 s with three trials with intertrial intervals of 10 min. The hot plate test was performed before SNI/sham surgery and 29 d postsurgery. Experiments were performed by a researcher blind to treatment and genotype.

### Anxiety-like behavioral tests

The elevated plus maze (EPM) and the open field test (OFT) were used to assess anxiety-like behavior in all mice (Descalzi et al., 2017). Briefly, the EPM apparatus consists of two open arms (29 cm) and two arms enclosed by walls (29 cm), and mice were placed in the center of the EPM and allowed to freely explore for 5 min. Tests were recorded and analyzed using the Noldus EthoVision XT software. Percent time spent exploring open arms (relative to total exploration of open and closed arms) were compared. Similarly, the OFT apparatus consists of a white square box (45 cm × 45 cm × 39 cm) in which mice are initially placed in one of the four corners and allowed to explore the field freely for 5 min. Percent time spent in the center (relative to total exploration of center and borders) was compared between cohorts. Experiments were performed 30 d (EPM) and 31 d (OFT) after sham or SNI surgery, by a researcher blind to treatment and genotype.

### qRT-PCR

RNA was extracted from the anterior cingulate cortex (ACC) tissue of mice using the RNeasy Mini Kit (Qiagen, 74104) and quantified with the NanoDrop (ND-2000, Thermo Fisher Scientific). Reverse transcription of RNA to cDNA was performed following the QuantiTect Reverse Transcription Kit protocol (Qiagen, 205313). After cDNA synthesis, qPCR was carried out using TaqMan Gene Expression Assays: FAM assay (catalog #4331182) for the *Becn1* gene (ID Mm01265461_m1) and VIC assay (catalog #4448484) for GAPDH (ID Mm99999915_g1) as the control. The plate was prepared with TaqMan Fast Advanced Master Mix (catalog #4444556). Data were quantified and analyzed using the Thermo Fisher Scientific Applied Biosystems QuantStudio software.

### Statistics

#### Statistical analysis

Data are expressed as mean ± SEM. Three-way repeated measures (RM), three-way, and one-way ANOVAs were used. The Greenhouse–Geisser correction was used for RM tests if the variance between groups differed. Where appropriate, Tukey's multiple-comparisons tests were employed. The normality of parametric datasets was confirmed by the D’Agostino and Shapiro–Wilk normality tests. All tests were two-tailed and conducted using GraphPad PRISM 10.3.1 software. Data were considered significant when *p* < 0.05. Statistical analyses can be found in Table 1.

**Table 1.** Statistical analyses for the data in Figures 1–5

	Data structure	Type of test	Statistic	*p*-value
Figure 1*A*	Normal distribution	Three-way RM ANOVA		
Time			*F*_(3.347, 147.3)_ = 283.8	*p* < 0.001
(Female vs male)			*F*_(1, 44)_ = 2.288	*p* = 0.14
(WT vs BCL)			*F*_(1, 44)_ = 17.15	*p* < 0.001
Time × (female vs male)			*F*_(5, 220)_ = 1.081	*p* = 0.37
Time × (WT vs BCL)			*F*_(5, 220)_ = 3.895	*p* = 0.002
(Female vs male) × (WT vs BCL)			*F*_(1, 44)_ = 2.454	*p* = 0.12
Time × (female vs male) × (WT vs BCL)			*F*_(5, 220)_ = 1.172	*p* = 0.32
Figure 1*B*	Normal distribution	Three-way RM ANOVA		
Time			*F*_(4.139, 182.1)_ = 4.579	*p* = 0.001
(Female vs male)			*F*_(1, 44)_ = 0.08293	*p* = 0.77
(WT vs BCL)			*F*_(1, 44)_ = 1.423	*p* = 0.24
Time × (female vs male)			*F*_(5, 220)_ = 0.8589	*p* = 0.51
Time × (WT vs BCL)			*F*_(5, 220)_ = 0.6759	*p* = 0.64
(Female vs male) × (WT vs BCL)			*F*_(1, 44)_ = 0.1692	*p* = 0.68
Time × (female vs male) × (WT vs BCL)			*F*_(5, 220)_ = 0.1891	*p* = 0.97
Figure 1*C*	Normal distribution	Three-way RM ANOVA		
Time			*F*_(5, 220)_ = 4.380	*p* < 0.001
(Female vs male)			*F*_(1, 44)_ = 0.01810	*p* = 0.89
(WT vs BCL)			*F*_(1, 44)_ = 0.05027	*p* = 0.82
Time × (female vs male)			*F*_(5, 220)_ = 1.053	*p* = 0.39
Time × (WT vs BCL)			*F*_(5, 220)_ = 0.1713	*p* = 0.97
(Female vs male) × (WT vs BCL)			*F*_(1, 44)_ = 2.059	*p* = 0.16
Time × (female vs male) × (WT vs BCL)			*F*_(5, 220)_ = 1.059	*p* = 0.38
Figure 1*D*,*E*	Normal distribution	Three-way RM ANOVA		
Time			*F*_(4, 176)_ = 55.66	*p* < 0.001
(Female vs male)			*F*_(1, 44)_ = 0.1896	*p* = 0.67
(WT vs BCL)			*F*_(1, 44)_ = 37.92	*p* < 0.001
Time × (female vs male)			*F*_(4, 176)_ = 1.207	*p* = 0.31
Time × (WT vs BCL)			*F*_(4, 176)_ = 0.2075	*p* = 0.93
(Female vs male) × (WT vs BCL)			*F*_(1, 44)_ = 8.123	*p* = 0.007
Time × (female vs male) × (WT vs BCL)			*F*_(4, 176)_ = 0.2403	*p* = 0.92
Figure 1*F*	Normal distribution	One-way ANOVA	*F*_(3, 76)_ = 3.746	*p* = 0.01
Figure 2*A*	Normal distribution	Three-way RM ANOVA		
Sex			*F*_(1, 90)_ = 18.70	*p* < 0.001
(WT vs BCL2)			*F*_(1, 90)_ = 1.734	*p* = 0.191
(Sham vs SNI)			*F*_(1, 90)_ = 0.07338	*p* = 0.787
Sex × (WT vs BCL2)			*F*_(1, 90)_ = 7.799	*p* = 0.006
Sex × (sham vs SNI)			*F*_(1, 90)_ = 0.6937	*p* = 0.407
(WT vs BCL2) × (sham vs SNI)			*F*_(1, 90)_ = 0.2768	*p* = 0.600
Sex × (WT vs BCL2) × (sham vs SNI)			*F*_(1, 90)_ = 0.01009	*p* = 0.920
Figure 2*B*	Normal distribution	Three-way RM ANOVA		
Sex			*F*_(1, 96)_ = 1.457	*p* = 0.230
(WT vs BCL2)			*F*_(1, 96)_ = 3.684	*p* = 0.058
(Sham vs SNI)			*F*_(1, 96)_ = 135.6	*p* < 0.001
Sex × (WT vs BCL2)			*F*_(1, 96)_ = 5.630	*p* = 0.020
Sex × (sham vs SNI)			*F*_(1, 96)_ = 0.03694	*p* = 0.848
(WT vs BCL2) × (sham vs SNI)			*F*_(1, 96)_ = 1.294	*p* = 0.258
Sex × (WT vs BCL2) × (sham vs SNI)			*F*_(1, 96)_ = 0.4683	*p* = 0.495
Figure 3*A*	Normal distribution	Three-way ANOVA		
Sex			*F*_(1, 79)_ = 3.007	*p* = 0.087
(WT vs BCL2)			*F*_(1, 79)_ = 8.180	*p* = 0.005
(Sham vs SNI)			*F*_(1, 79)_ = 23.96	*p* < 0.001
Sex × (WT vs BCL2)			*F*_(1, 79)_ = 1.719	*p* = 0.194
Sex × (sham vs SNI)			*F*_(1, 79)_ = 0.04524	*p* = 0.832
(WT vs BCL2) × (sham vs SNI)			*F*_(1, 79)_ = 0.1233	*p* = 0.726
Sex × (WT vs BCL2) × (sham vs SNI)			*F*_(1, 79)_ = 2.325	*p* = 0.131
Figure 3*B*	Normal distribution	Three-way ANOVA		
Sex			*F*_(1, 79)_ = 206.0	*p* < 0.001
(WT vs BCL2)			*F*_(1, 79)_ = 33.22	*p* < 0.001
(Sham vs SNI)			*F*_(1, 79)_ = 2.709	*p* = 0.104
Sex × (WT vs BCL2)			*F*_(1, 79)_ = 12.23	*p* < 0.001
Sex × (sham vs SNI)			*F*_(1, 79)_ = 6.929	*p* = 0.010
(WT vs BCL2) × (sham vs SNI)			*F*_(1, 79)_ = 2.733	*p* = 0.102
Sex × (WT vs BCL2) × (sham vs SNI)			*F*_(1, 79)_ = 0.2839	*p* = 0.596
Figure 4*A*	Normal distribution	Three-way ANOVA		
Sex			*F*_(1, 81)_ = 4.726	*p* = 0.033
(WT vs BCL2)			*F*_(1, 81)_ = 0.4593	*p* = 0.500
(Sham vs SNI)			*F*_(1, 81)_ = 11.41	*p* = 0.001
Sex × (WT vs BCL2)			*F*_(1, 81)_ = 0.9078	*p* = 0.344
Sex × (sham vs SNI)			*F*_(1, 81)_ = 0.6091	*p* = 0.437
(WT vs BCL2) × (sham vs SNI)			*F*_(1, 81)_ = 6.308	*p* = 0.014
Sex × (WT vs BCL2) × (sham vs SNI)			*F*_(1, 81)_ = 0.1998	*p* = 0.656
Figure 4*B*	Normal distribution	Three-way ANOVA		
Sex			*F*_(1, 81)_ = 15.31	*p* < 0.001
(WT vs BCL2)			*F*_(1, 81)_ = 0.4593	*p* = 0.500
(Sham vs SNI)			*F*_(1, 81)_ = 0.4492	*p* = 0.505
Sex × (WT vs BCL2)			*F*_(1, 81)_ = 0.6998	*p* = 0.405
Sex × (sham vs SNI)			*F*_(1, 81)_ = 1.336	*p* = 0.251
(WT vs BCL2) × (sham vs SNI)			*F*_(1, 81)_ = 0.5748	*p* = 0.451
Sex × (WT vs BCL2) × (sham vs SNI)			*F*_(1, 81)_ = 0.03412	*p* = 0.854
Figure 5	Normal distribution	Three-way ANOVA		
Sex			*F*_(1, 35)_ = 4.067	*p* = 0.051
(WT vs BCL2)			*F*_(1, 35)_ = 0.06186	*p* = 0.805
(Sham vs SNI)			*F*_(1, 35)_ = 0.3581	*p* = 0.553
Sex × (WT vs BCL2)			*F*_(1, 35)_ = 3.708	*p* = 0.062
Sex × (sham vs SNI)			*F*_(1, 35)_ = 3.467	*p* = 0.071
(WT vs BCL2) × (sham vs SNI)			*F*_(1, 35)_ = 0.1879	*p* = 0.667
Sex × (WT vs BCL2) × (sham vs SNI)			*F*_(1, 35)_ = 4.557	*p* = 0.040

## Results

### Disruptions of BECLIN-1 enhance mechanical allodynia caused by nerve injury

**Figure 1.**
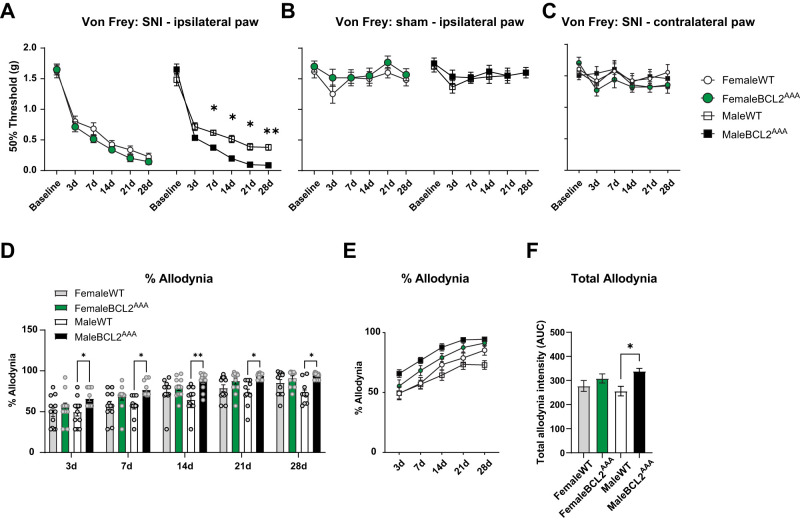
Disruption of BECLIN-1 enhances mechanical pain hypersensitivity in male but not female mice in a mouse model of neuropathic pain. ***A***, Von Frey measures expressed in grams of injured (ipsilateral) paw from female and male WT and *Bcl2^AAA^* mice exposed to SNI and (***B***) sham surgery. Male *Bcl2^AAA^* mice showed significantly lower mechanical thresholds at 7, 14, 21, and 28 d postinjury, compared with WT SNI mice, whereas female *Bcl2^AAA^* mice showed similar mechanical thresholds compared with WT SNI mice. No differences were observed in sham-treated mice. ***C***, Von Frey measures of noninjured (contralateral) paw from female and male WT and *Bcl2^AAA^* mice exposed to SNI surgery. ***D***, Quantification as percentage of allodynia by SNI (% allodynia) across time. Male *Bcl2^AAA^* mice displayed significantly greater levels of SNI-induced allodynia compared with WT mice, whereas no differences were detected between female *Bcl2^AAA^* and WT mice. ***E***, % allodynia in female and male *Bcl2^AAA^* and WT mice. ***F***, Quantification of total percent allodynia (area under the curve, AUC, from ***E***). Three-way RM and one-way ANOVA, *n* = 12 per group; *p < 0.05, **p < 0.01.

To investigate the role of the BECLIN-1 protein in relation to nociceptive behavioral responses in a model of chronic neuropathic pain, we assessed pain-related behavior expressed by female and male WT and *Bcl2^AAA^* mice. Using von Frey filaments, we measured mechanical thresholds before and after SNI or sham procedures and performed a three-way ANOVA to determine if there were any differences. We found a significant effect of time, a significant difference between WT and *Bcl2^AAA^* mice, and a significant interaction between time and genotype (Table 1). Interestingly, although no significant difference was detected prior to injury (baseline) between all mice, male *Bcl2^AAA^* mice exposed to SNI showed significantly lower mechanical thresholds compared with male WT mice at 7, 14, 21, and 28 d postinjury, whereas female *Bcl2^AAA^* SNI mice showed similar levels of mechanical thresholds compared with female WT SNI mice. Surprisingly, we did not detect a significant interaction between sex and genotype (Fig. 1*A*). No differences were observed among all groups exposed to sham procedures (Fig. 1*B*) or in the contralateral, noninjured paw (Fig. 1*C*). Moreover, when we assessed the extent of allodynia expressed by *Bcl2^AAA^* SNI mice, we found that male *Bcl2^AAA^* SNI mice showed significantly higher levels of allodynia across all time points postinjury, whereas female *Bcl2^AAA^* mice showed similar allodynia levels compared with female WT mice. Accordingly, a significant interaction was found between sex and genotype (Fig. 1*D*–*F*).

**Figure 2.**
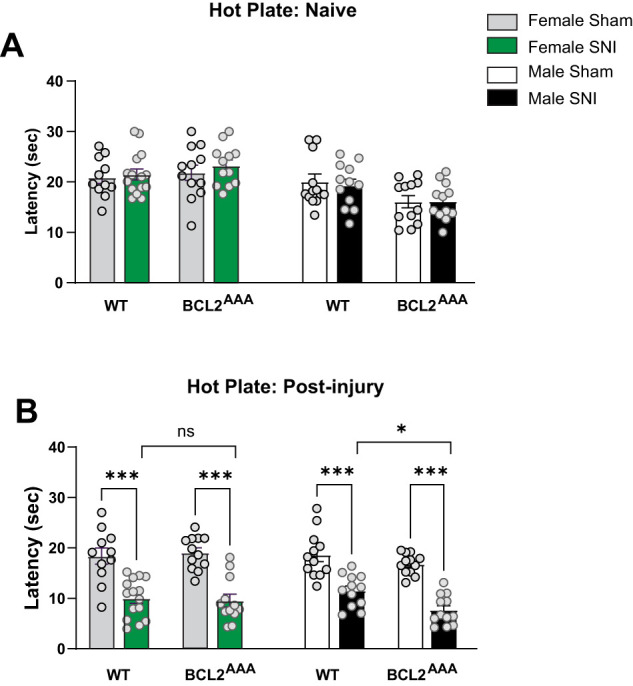
Disruption of BECLIN-1 enhances thermal pain hypersensitivity in male but not female *Bcl2^AAA^* mice. ***A***, Hot plate latency measures in female and male WT and *Bcl2^AAA^* naive mice; no differences were observed. ***B***, Hot plate latency measures in female and male WT and *Bcl2^AAA^* mice exposed to SNI and sham surgery. Female and male WT and *Bcl2^AA^* mice exposed to SNI showed significantly shorter response latencies compared with sham controls. Male, but not female, *Bcl2^AAA^* mice showed significantly shorter response latencies compared with WT SNI mice. Three-way ANOVA, *n* = 10–15 per group; *p < 0.05, ***p < 0.001.

We also assessed the effects of SNI on thermal thresholds in female and male WT and *Bcl2^AAA^* mice using the hot plate test (50 ± 0.2°C). As can be seen in Figure 2*A*, female and male WT and *Bcl2^AAA^* naive mice, that is, prior to receiving SNI or sham surgery, showed similar response latencies in the hot plate test. Surprisingly, a three-way ANOVA showed that there was a significant effect of sex, with a significant interaction between sex and genotype, whereby female WT and *Bcl2^AAA^* naive mice showed significantly longer latencies than male *Bcl2^AAA^* naive mice (Table 1). Twenty-nine days post SNI or sham surgery, both female and male WT and *Bcl2^AAA^* mice exposed to SNI showed a significant reduction in their response latencies compared with sham controls (Fig. 2*B*). Consistent with our findings of mechanical allodynia levels, a significant interaction between sex and genotype was also noted, whereby male *Bcl2^AAA^* SNI mice, but not female *Bcl2^AAA^* SNI mice, showed significantly larger reductions in their response latency when compared with WT mice in the SNI cohort (Table 1).

### Disruptions of BECLIN-1 do not affect SNI-induced changes in anxiety-like behaviors

**Figure 3.**
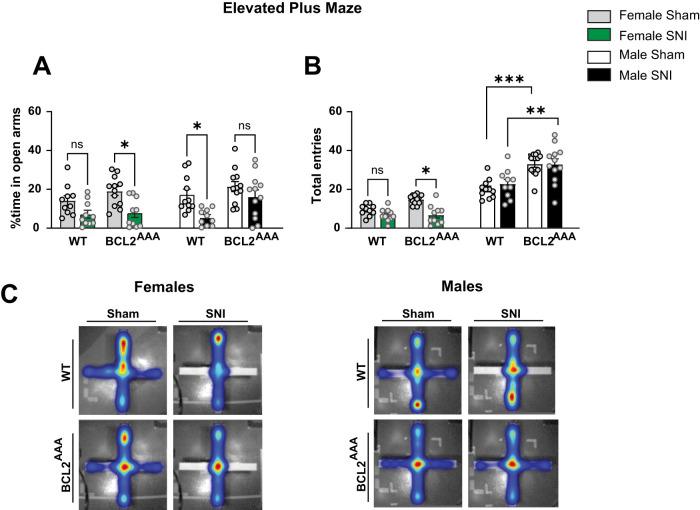
Disruption of BECLIN-1 prevents SNI-induced increases in anxiety-like behavior in the EPM in male mice. ***A***, % time spent exploring the open arms of an EPM by SNI and sham-treated female and male WT and *Bcl2^AAA^* mice. Whereas male WT mice showed a significant reduction in open arm exploration compared with sham-treated mice, male *Bcl2^AAA^* mice showed no reductions in open arm exploration. ***B***, Total entries displayed by mice in ***A***. ***C***, Representative heat maps of female (left) and male (right) mice exploration during EPM tests. Three-way ANOVA, *n* = 10–12 per group; *p < 0.05.

**Figure 4.**
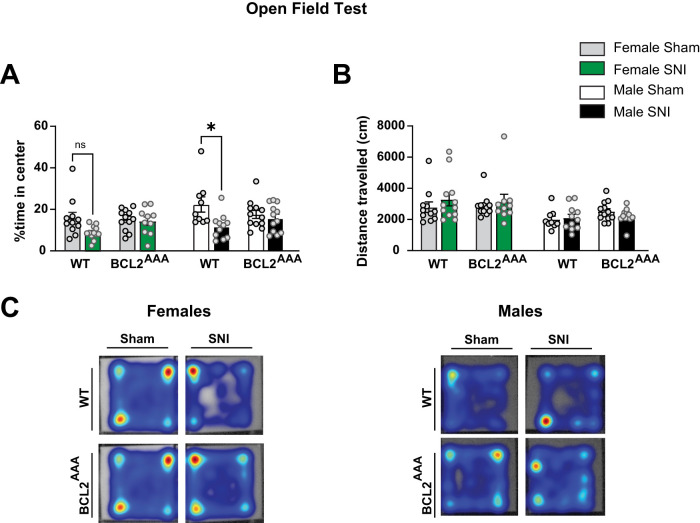
Disruption of BECLIN-1 prevents SNI-induced increases in anxiety-like behavior in the open field test in male mice. ***A***, % time spent in the center area of an open field arena by female and male WT and *Bcl2^AAA^* SNI and sham-treated mice. Male SNI *Bcl2^AAA^* mice showed similar exploration time of the center area in the open field test compared with sham-treated controls. ***B***, Total distance traveled by male WT and *Bcl2^AAA^* mice in an OFT 30–31 d after SNI and sham surgery. ***C***, Representative heat maps of female (left) and male (right) mouse exploration during open field tests. Three-way ANOVA, *n* = 10–12 per group; *p < 0.05.

Reflecting comorbidities observed in humans with chronic pain (Bair et al., 2008; Choinière et al., 2010), mouse models of neuropathic injury result in nociceptive hypersensitivity and increases in anxiety-like and depression-like behaviors in both female and male mice (Yalcin et al., 2011; Chen et al., 2013; Terzi et al., 2014; Koga et al., 2015; Descalzi et al., 2017; Sellmeijer et al., 2018). We thus next sought to determine if disruptions in BECLIN-1 signaling can affect SNI-induced increases in anxiety-like behaviors by using the elevated plus maze (EPM) and open field tests (OFT) on Days 30–31 after SNI or sham procedures. As expected, in the EPM, we found that male WT SNI mice exhibit a significant reduction in the percent time spent exploring the open arms compared with male WT sham controls, and observed a strong, albeit nonsignificant, trend of reduced open arm exploration in female WT SNI mice compared with female WT sham controls (Fig. 3*A*, Table 1). Remarkably, in contrast to male WT mice, male *Bcl2^AAA^* SNI and sham mice did not show any differences in time spent exploring the open arms, whereas female *Bcl2^AAA^* SNI mice showed a significant reduction compared with female *Bcl2^AAA^* sham mice. Surprisingly, we did not detect a significant interaction between sex and genotype. A significant interaction between sex and genotype was observed in total EPM entries, whereby female *Bcl2^AAA^* SNI mice showed a significant reduction in entries compared with female WT SNI mice, whereas male WT SNI and male *Bcl2^AAA^* SNI mice showed similar levels (Fig. 3*B*). In accordance with our EPM findings, in the OFT, we found that male WT SNI mice spent a significantly less amount of time in the center area compared with their male WT sham counterparts and a strong, but not significant, trend in female WT SNI mice showing reduced time in the center area, whereas no differences were observed between male *Bcl2^AAA^* SNI and sham mice in the OFT (Fig. 4*A*, Table 1). Neither WT nor *Bcl2^AAA^* (female or male) mice exposed to SNI showed any changes in distance traveled, and although no differences were detected between genotypes, a significant main effect of sex was noted, whereby both female WT and *Bcl2^AAA^* SNI and sham-treated mice explored greater distances than male WT and *Bcl2^AAA^* SNI and sham-treated mice (Fig. 4*B*).

**Figure 5.**
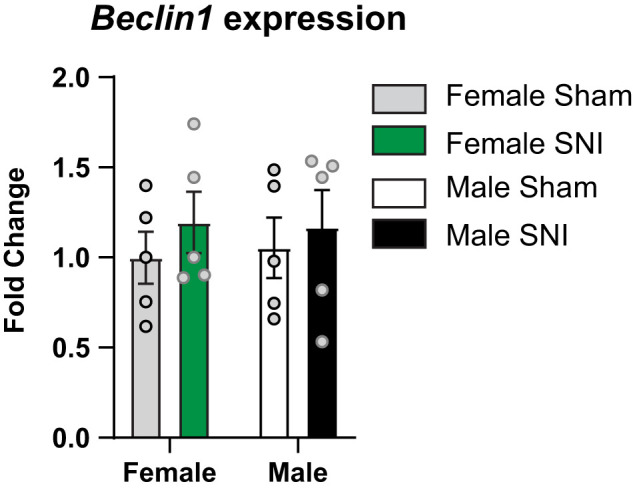
*Beclin-1* expression in the anterior cingulate cortex does not change in response to SNI. No differences in expression levels of *Beclin-1* RNA were observed in the ACC between groups. RNA expression levels were quantified through qRT-PCR. ACC tissue samples were collected from female and male WT SNI and sham-treated mice 31 d after SNI or sham procedures. One-way ANOVA, *n* = 5 per group.

The effects of BECLIN-1 disruption on nociceptive thresholds and anxiety-like behaviors suggest the possibility that BECLIN-1 activity is altered by neuropathic pain. To determine this, we performed qRT-PCR to assess mRNA expression levels of *Beclin-1* in the anterior cingulate cortex (ACC), a brain region that has been well-documented to display plasticity in mouse models of chronic pain and is critical for the expression of pain hypersensitivity and increases in anxiety-like behavior in mouse models of neuropathic pain (Zhuo, 2008; Li et al., 2010; Koga et al., 2015; Fillinger et al., 2018; Song et al., 2024). We found that female and male mice exposed to SNI showed similar levels of *Beclin-1* compared with sham-treated controls (Fig. 5, Table 1).

## Discussion

In this study, we investigated whether the transgenic disruption of stimulus-induced autophagy, by targeting the BECLIN-1 protein, would affect the development of chronic neuropathic pain and associated anxiety-like behavior in female and male mice.

We found a sex-specific role for BECLIN-1 in nerve injury-induced nociceptive hypersensitivity, whereby male, but not female, *Bcl2^AAA^* mice exposed to SNI showed significantly enhanced mechanical allodynia compared with WT SNI cohorts, which was evident as early as 3 d postinjury and lasting at least until Day 28. Similarly, male, but not female, *Bcl2^AAA^* mice exposed to SNI showed significantly enhanced thermal nociceptive hypersensitivity compared with WT male mice exposed to SNI at 29 d postinjury. Accordingly, a recent study found that disruptions of BECLIN-1 augmented mechanical nociceptive hypersensitivity in a mouse model of chronic inflammatory pain, and as with our findings, these effects were only observed in male and not female mice (Tam et al., 2024). Moreover, these findings support previous observations using male mice, whereby spinal nerve ligation was found to decrease markers of autophagy in the spinal cord at 7 d postinjury (Berliocchi et al., 2011). Together, these data indicate that activation of the BECLIN-1 protein stimulus-induced autophagy pathway might help attenuate nerve injury-induced allodynia and hyperalgesia, such that disruptions of this process promote increases in nociceptive hypersensitivity. Remarkably, our data indicate that this effect is sex-dependent, whereby female *Bcl2^AAA^* SNI mice did not display any differences in nociceptive responses compared with female WT SNI mice. Such findings display the potential for sex-specific functions of BECLIN-1 protein and indicate that reductions in autophagy promote pain hypersensitivity in male mice. Notably, autophagy is a key activator of inflammatory responses, and a loss of function in BECLIN-1 has been found to dysregulate immune responses, including microglia release of IL1-β, a cytokine which has consistently been implicated in mouse neuropathic pain models (Scholz and Woolf, 2007; Houtman et al., 2019; Yi et al., 2021; Xu et al., 2023). This suggests that one possible mechanism through which BECLIN-1 disruption alters nociceptive hypersensitivity and anxiety-like behaviors in male mice is through altering injury-related immune responses, including the release of IL-1β. Future studies should thus investigate whether IL-1β is disrupted in *Bcl2^AAA^* SNI mice, and if these sex differences extend to nerve injury-induced IL-1β release as seen previously with reduced autophagy models (Houtman et al., 2019). Notably, while male *Bcl2^AAA^* SNI mice showed enhanced nociceptive hypersensitivity in the injured paw, their right paws showed no significant difference, indicating that disruptions in BECLIN-1 do not cause a systemic hypersensitivity of nociceptive responses, unlike what has been previously observed with other transgenic lines that show increases in nociceptive hypersensitivity (Descalzi et al., 2012). Another possibility is that the enhanced nociceptive hypersensitivity observed in *Bcl2^AAA^* SNI mice is due to an unregulated inflammatory response due to the BECLIN-1 protein being absent in maintaining healing and repair of the injury (Huang et al., 2016).

Remarkably, whereas BECLIN-1 disruption enhanced nociceptive hypersensitivity in male mice exposed to SNI, we found that it conversely blocked SNI-induced increases in anxiety-like behaviors. The effects of BECLIN-1 disruption in pain-induced anxiety-like behavior in female mice are harder to interpret, as female WT mice did not show statistically significant increases in anxiety-like behavior, making comparisons difficult. Moreover, although female *Bcl2^AAA^* SNI mice showed significantly less open arm exploration compared with *Bcl2^AAA^* sham mice in the EPM test, *Bcl2^AAA^* SNI mice also showed significantly less total entries into open or closed arms in general, indicating a strong reduction in exploration, which may not be anxiety-related in nature. Our data from male mice however strongly indicate that the role of autophagy in neuropathic pain extends beyond pain hypersensitivity and also plays a contrasting role in the affective component of pain in male mice. Specifically, these findings show that reductions in autophagy prevent the development of neuropathic pain-induced increases in anxiety-like behavior in male mice. Similar findings have been observed in male rats treated with intrahippocampal injections of amyloid β1–42 peptide, whereby treatments that reduced anxiety-like behaviors corresponded with decreases in autophagy within the hippocampus (Song et al., 2017). These findings are in contrast with previous observations in male rats, which found that decreases in macroautophagy in the prelimbic cortex were associated with neuropathic pain-induced increases in anxiety (Fu et al., 2024). This could be partly due to the use of different species and may also underscore that our observations are not mediated by the prelimbic cortex. Notably, we found that *Beclin-1* expression was not altered in the ACC of female or male mice exposed to SNI, further highlighting differences in our models. Similarly, it was recently shown that neither chronic inflammatory pain nor SNI altered expression levels of BECLIN-1 protein in the mouse dorsal horn (Tam et al., 2024). We assessed expression in the ACC as it has been repeatedly shown to display plasticity in neuropathic pain models and has a critical role in the expression of pain hypersensitivity and increases in anxiety-like behavior in mouse models of neuropathic pain (Zhuo, 2008; Li et al., 2010; Koga et al., 2015); however, a broader cortical analysis of *Beclin-1* expression may highlight fundamental autophagy-related mechanisms that were missed by our analysis. Future studies should investigate this possibility. Increases in anxiety-like behavior resulting from neuropathic pain in mice have been observed widely (Yalcin et al., 2011; Guida et al., 2020), contributing to the affective component of pain. While it remains difficult to study the emotional aspects of pain in nonhuman models, human clinical studies report increased incidences of anxiety, depression, and other mood-related symptoms, including a reduction in quality of life (Bair et al., 2008; Guida et al., 2020). The protective effect of BECLIN-1 disruption in male SNI mice against anxiodepressive behavior development has not previously been reported.

It is important to note that the effect sizes of our findings are rather small, and this should be considered when reflecting on the overall impact and generalizability of our current findings. Importantly, we found that whereas disruptions of BECLIN-1 did not affect thermal hypersensitivity in mice exposed to neuropathic pain, it did appear to exert an effect on sham control animals. We also observed that whereas disruptions of BECLIN-1 prevented pain-induced increases in anxiety-like behavior, there is an effect of BECLIN-1 disruption on exploratory behavior, suggesting that anxiety-related effects of BECLIN-1 disruption may not be limited to pain.

In conclusion, our study demonstrated the role of BECLIN-1 in neuropathic pain-associated mechanical and thermal nociceptive hypersensitivity. Moreover, our data reveal that this is a sex-specific phenotype as its impaired function led to increased pain hypersensitivity in male mice but not in female mice. Notably, male mice with disruptions in BECLIN-1 that suffered from chronic neuropathic pain did not demonstrate the robust increases in anxiety-like behavior that we observed in male WT mice, suggesting that decreases in autophagy reduce anxiety-like behavior. Our findings thus reveal a sex-specific bidirectional role of BECLIN-1 in pain and anxiety-like behavior. Furthermore, due to the sex-specific nature of the responses, our data indicate that pain may differ in clinical phenotypes and management in males as compared with females. Further studies need to be conducted to investigate the molecular mechanisms that govern pain hypersensitivity and anxiety-like behavior, in both sexes.

## Synthesis

Reviewing Editor: Jibran Khokhar, Western University Department of Anatomy and Cell Biology Decisions are customarily a result of the Reviewing Editor and the peer reviewers coming together and discussing their recommendations until a consensus is reached. When revisions are invited, a fact-based synthesis statement explaining their decision and outlining what is needed to prepare a revision will be listed below. The following reviewer(s) agreed to reveal their identity: Anna Taylor.

In this study, the authors present evidence that BECLIN1, a regulatory protein involved in autophagy, plays a role in sensory and affective symptoms of neuropathic pain. Using a transgenic knockout mouse model for BECLIN1, they demonstrate that male mice exhibit enhanced pain sensitivity but reduced pain-associated anxiety in a model of neuropathic pain induced by spared nerve injury (SNI). Interestingly, female mice did not show similar changes in pain sensitivity when BECLIN1 was absent. The authors conclude that this study reveals significant sex differences in how BECLIN1 mediates neuropathic pain. This research extends previous studies by examining these processes in both sexes, adding valuable insights into the field. The experimental design is well-constructed, and the manuscript is clearly written. However, there are major and minor concerns regarding the statistical analysis and other aspects of the study.

Major Concerns

1. Sex-Based Statistical Analysis: The primary hypothesis is whether deletion of BECLIN1 affects pain symptoms differently between males and females. However, no statistical tests were performed to determine if there is a significant effect of sex. The authors only show significant differences between WT and KO in males and not in females, but this is not sufficient to conclude a significant effect of sex. A statistical analysis that includes sex as a factor is necessary.

2. Appropriate Strain Controls: It is unclear if the appropriate strain controls were used. The comparison between KO mice and C57 mice would be problematic if they are on different backgrounds (C57 vs B6.129X1). Littermate controls should be used to ensure the validity of the statistical comparisons.

3. BECLIN1 Expression Analysis: BECLIN1 expression was not assessed at the mRNA or protein level. It is crucial to know when and where BECLIN1 is expressed after SNI, and whether the changes in pain outcomes mirror expression patterns. The changes in anxiety-like behaviors also suggest a potential mechanism involving altered BECLIN1 expression in the brain or a systems-level effect, which needs to be studied. The authors mention an altered immune response but do not assess changes in inflammatory mediators.

4. Consistency in Measurement Units: The authors show changes in mechanical thresholds as a percent change but use latency (seconds) for thermal responses. It is necessary to show the von Frey data in grams, and a percent change in thermal thresholds may be needed if there is a baseline effect in mechanical nociception.

Minor Concerns

1. Baseline Sensitivity: Differences in baseline mechanical thresholds between WT and KO (no pain) are not indicated. It is important to know if there were significant differences in mechanical thresholds before SNI induction.

2. Clarification in Methods: Under "Neuropathic Pain Model", the sentence "Briefly, mice were induced..." should be clarified to "...anesthesia was induced...".

3. Fig 3B Labeling: There is a labeling issue on the X-axis of Fig 3B.

4. Contralateral Paw Thresholds: The discussion refers to contralateral paw thresholds, but no data indicates these were measured. Testing contralateral pain thresholds would inform whether BECLIN1 knockout affects pain sensitivity systemically or is limited to the injured site.

Additional Comments

1. Reference to Related Work: A manuscript by Tam et al., published in Cell Reports in 2024, shows a sex-dependent effect for BECLIN1 in inflammatory pain. This should be cited in the resubmission.

2. Repetition and Clarity in Text: There is considerable repetition in the text, and extensive editing is required in the results section to clarify what the authors are comparing, particularly for the thermal hypersensitivity and anxiety behavior data.

3. Reference Formatting: The references need to be edited for consistent formatting.

4. Figure Legends and Labels: Fig 3B has extra words on the X-axis, and it is unclear which mice are injured and which are baseline/sham in Fig 3. The legends are inappropriate and need revision.
